# Anaerobic microplate assay for direct microbial conversion of switchgrass and Avicel using *Clostridium thermocellum*

**DOI:** 10.1007/s10529-017-2467-2

**Published:** 2017-11-09

**Authors:** Gbekeloluwa B. Oguntimein, Miguel Rodriguez, Alexandru Dumitrache, Todd Shollenberger, Stephen R. Decker, Brian H. Davison, Steven D. Brown

**Affiliations:** 1 Morgan State University, Baltimore, MD USA; 2 Biosciences Division, Oak Ridge National Laboratory, Oak Ridge, TN USA; 3 BioEnergy Science Center, National Laboratory, Oak Ridge, TN USA; 4 National Renewable Energy Laboratory, Golden, CO USA; 5 Present Address: LanzaTech Inc, 8045 Lamon Ave, Suite 400, Skokie, IL 60077 USA

**Keywords:** Bioethanol, Consolidated bioprocessing, High throughput screening, Lignocellulosic biomass, Microplate assay

## Abstract

**Objective:**

To develop and prototype a high-throughput microplate assay to assess anaerobic microorganisms and lignocellulosic biomasses in a rapid, cost-effective screen for consolidated bioprocessing potential.

**Results:**

*Clostridium thermocellum* parent Δ*hpt* strain deconstructed Avicel to cellobiose, glucose, and generated lactic acid, formic acid, acetic acid and ethanol as fermentation products in titers and ratios similar to larger scale fermentations confirming the suitability of a plate-based method for *C. thermocellum* growth studies. *C. thermocellum* strain LL1210, with gene deletions in the key central metabolic pathways, produced higher ethanol titers in the Consolidated Bioprocessing (CBP) plate assay for both Avicel and switchgrass fermentations when compared to the Δ*hpt* strain.

**Conclusion:**

A prototype microplate assay system is developed that will facilitate high-throughput bioprospecting for new lignocellulosic biomass types, genetic variants and new microbial strains for bioethanol production.

## Introduction

The global rise in energy demand, unstable and limited petroleum resources, and environmental concerns have led to the development of sustainable resources for bioethanol production. According to the National Council of State Legislatures (NCSL), lawmakers in 26 states in the USA passed legislation in 2005–2006 to encourage the creation of biofuels-related companies and authorized millions of dollars in grants (both to universities and companies) to conduct research and create a commercial sector to distribute this alternative energy source (Pellerito 2006). Lignocellulosic biomass, such as wood, grasses, forest and agricultural residues, has been highlighted as having great potential for bioenergy production since it is inedible and the most abundant resource in the world. As a promising energy crop, Miscanthus (Lee and Kuan 2015; Yoo et al. 2016), Poplar (Sannigrahi et al. 2010) and switchgrass (David and Ragauskas 2010) have received attention due to their advantages including high yield of biomass per unit planted area, ability to grow without pesticides or fertilizers, low maintenance and long lifespan. Switchgrass (*Panicum viragatum*), a perennial warm season C4 grass and a dominant species of the remnant tall grass prairies, has gained increasing attention for use in biofuel production because relatively low energy input is required for production and a high biomass yield. One of the advantages of switchgrass is that it can produce five times more renewable energy than nonrenewable energy consumed, while the greenhouse gas emissions from ethanol produced from switchgrass are 94% less than those from gasoline. In the USA switchgrass has a yield of 5 tons/acre and an annual yield up to 11.1 Mg/ha. Together with miscanthus (Miscanthus × giganteus), switchgrass has been identified as one of the better feedstocks for bioethanol production (Wang et al. 2012).

Rapid high throughput (HTP) determination of the potential of lignocellulosic biomass feedstocks is an important enabling technology for the development of cellulosic biofuels. HTP screening allows the investigation of large sample sets to be undertaken with increased speed and cost effectiveness. An assay used to determine bioethanol production from large numbers of lignocellulosic biomasses (LCBs) must be robust, rapid, and easy to perform, and must use modest amounts of the samples to be tested. Over the past 15 years or so, various labs have devised screening protocols for analyzing LCB digestion, evaluating the efficacy of enzymes, recalcitrance of the substrate, composition of the biomass, or the conversion potential of microbial strains with throughputs ranging from a couple of dozen to thousands per day depending on the scale and assay type. For example, Selig et al. (2010) developed a combined HTP thermochemical pretreatment and enzyme hydrolysis assay (i.e. sugar release) assay for evaluating thousands of biomass samples for recalcitrance. The group also modified protocols for providing carbohydrate compositional analyses (Selig et al. 2011) along with insights into lignin composition (Sykes et al. 2009). Elliston et al. (2015) reported a methodology to enable solid lignocellulosic substrates to be simultaneously saccharified and fermented by yeast fermented in a 96-well plate scale, facilitating HTP screening of ethanol production, whilst maintaining repeatability similar to that achieved at a larger scale. The goal of this study was to develop and prototype a high-throughput microplate assay to assess anaerobic microorganisms and lignocellulosic biomasses in a rapid, cost-effective screen for consolidated bioprocessing potential (CBP, i.e. to deconstruct and ferment sugars from LCB in a single step with specialized microbes).

## Materials and methods

### Substrates

Crystalline cellulose (Avicel PH105) was obtained from FMC Corporation, Philadelphia, USA. Switchgrass used in this study was lowland cultivar Alamo switchgrass grown by the Samuel Roberts Noble Foundation in Ardmore, Oklahoma, milled and passed through a 20-mesh screen. All other chemicals were analytical grade.

### Microorganisms and growth conditions

Strains were used in this study: wild-type *Clostridium thermocellum* strain ATCC 27405 (Dumitrache, et al., 2016), strain LL1210 (Δ*hpt* Δ*hydG* Δ*ldh* Δ*pfl* Δ*pta*-*ack)* and its parent *C. thermocellum* DSM1313 Δ*hpt* (Tian et al. 2016). For batch bottle cultures, strains were grown in defined MTC medium, which was prepared anaerobically by mixing independently sterilized solutions. All medium components are reported here at final complete medium concentrations, per liter of ultrapure water. Initially, solution A (2.5 g Avicel, 0.001 g Resazurin) was sterilized by autoclaving for 30 min. Solution D (1 g MgCl_2_·6H_2_O, 0.2 g CaCl_2_·2H_2_O, 0.1 g FeCl_2_·4H_2_O, 1 g l-cysteine hydrochloride monohydrate) was also prepared fresh for each set of experiments and sterilized by autoclaving. The remaining solutions, sterilized by filtration, contained the following: solution B + M (2 g potassium citrate, 1.25 g citric acid monohydrate, 1 g Na_2_SO_4_, 1 g KH_4_PO_4_, 2.5 g NaHCO_3_, 5 g MOPS, solution C (1.5 g NH_4_Cl, 2 g urea), solution E (20 mg pyridoxamine dihydrochloride, 1 mg riboflavin, 1 mg nicotinamide, 0.5 mg lipoic acid, 4 mg 4-aminobenzoic acid, 4 mg d-biotin, 0.025 mg folic acid, 2 mg cyanocobalamin, 0.2 mg thiamine hydrochloride), and solution F (0.5 mg MnCl_2_·4H_2_O, 0.5 mg CoCl_2_·6H_2_O, 0.2 mg ZnSO4·7H_2_O, 0.05 mg CuSO_4_·5H_2_O, 0.05 HBO_3_, 0.05 mg Na_2_MoO_4_·2H_2_O, 0.05 mg NiCl_2_·6H_2_O, 10 mg citric acid monohydrate). Filter sterilized solutions (B + M, C, E, F) were prepared as concentrated stocks (25×, 50×, 50×, and 1000×, respectively). Medium composition for pH-controlled batch bioreactor cultures was similar, with the replacement in solution A of Avicel with *Populus* biomass (20 g/l on a dry basis) and the omission of MOPS from solution B + M (Dumitrache et al. 2017).

### Microplate loading

A Powdernium powder dispensing system (Symyx, Geneva, Switzerland) was used to dispense milled BESC switchgrass into 96 deep well microplates as shown. In addition, the system utilizes a modified balance (Sartorius LP330, Goettingen, Germany), which records the final weight dispensed into each well to 0.1 mg (Selig et al. 2010). Thermo Scientific Nunc 2 ml deepwell plates in 96-well format with shared-wall technology and Thermo Scientific Nunc 96-well cap mats were used in one configuration and VWR DeepWell Microplate DeepWell, polypropylene, 2 ml plate in 96-well format and VWR mat lids polyethylene mat lids (VWR Scientific, Radnor, PA) were also used. Forty-eight wells were filled with 1 g BESC switchgrass and the other 48 wells filled with 1 g Avicel. Rows A, B, and C were inoculated with the Δ*hpt* strain and EFG with strain LL1210. Rows D and H were inoculated with sterilized water. Plates were placed at a 45-degree angle on an orbital shaker (Cole Palmer Model 51300) set at 125 rpm. The shaker was held at 60 °C maintained in an anaerobic atmosphere (5% H_2_, 10% CO_2_, and 85% N_2_) inside a Coy vinyl glove bag (Coy Laboratories Products Inc., Grass Lake, MI).

### Analysis of fermentation products

Fermentation products were determined by HPLC using an Aminex HPX-87H column (Bio-Rad) at 60 °C with 5 mm H_2_SO_4_ as mobile phase at 0.5 ml/min. The eluate was monitored by a refractive index detector at 35 °C.

## Results and discussion

### Metabolite profile during fermentation

**Fig. 1:**
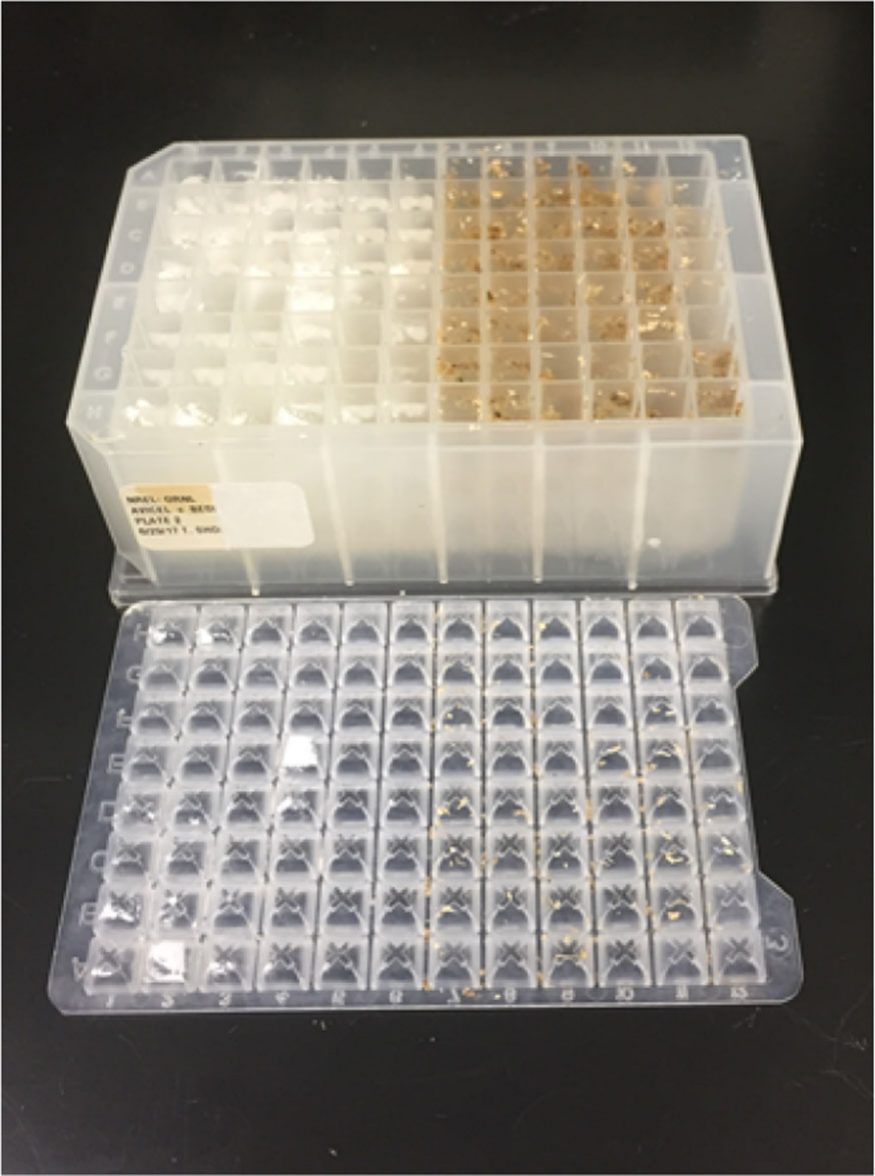
Example of 96-deep well plate loaded with avicel and switchgrass substrates

This study describes initial method development to enable high throughput (HTP) screening of lignocellulosic biomass (LCB) for Consolidated Bioprocessing (CBP). HTP has been developed for enzymatic hydrolyses on solid biomass but CBP on these materials has been carried out only at relatively low throughput in tube, bottle or bioreactor fermentation at the laboratory scale. CBP methodology on a solid substrate has not been investigated at much smaller HTP biomass loadings. The robotic loading of biomass or Avicel into 96-well plates provided accurate substrate loadings (Fig. 1), consistent with earlier studies (Selig et al. 2011).

**Fig. 2:**
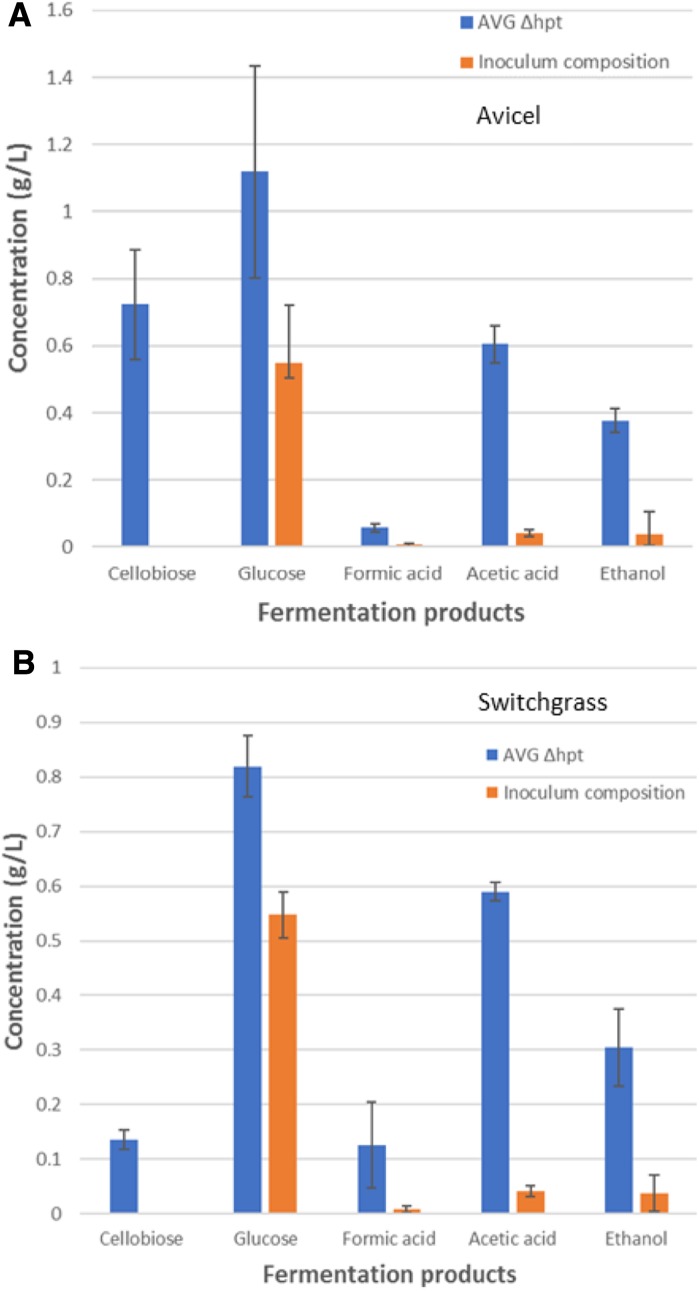
Fermentation products generated by *C. thermocellum* strain Δ*hpt* with **a** Avicel as the substrate, or **b** switchgrass as the substrate

*Clostridium thermocellum* parent Δ*hpt* strain deconstructed Avicel to cellobiose, glucose, and generated lactic acid, formic acid, acetic acid and ethanol as fermentation products (Fig. 2a) in titers and ratios seen in larger scale fermentations confirming the suitability of a plate-based method for *C. thermocellum* growth. The composition of the inoculum applied to the plate culture is shown, which indicates that growth and fermentation occurred in the microplate. *C. thermocellum* enzymes can continue to act on substrates in the absence of microbial growth, which is a possible reason for relatively higher carry over levels of glucose in inocula samples. To further assess the potential of the plate based method, CBP assays were conducted using switchgrass and a parent strain (Δ*hpt*) (Fig. 2b). The CBP assay results were similar to fermentations of avicel and switchgrass from earlier reports (Argyros et al. 2011; Yee et al. 2014; Tian et al. 2016; Dumitrache et al. 2016), further supporting a plate based CBP assay.

**Fig. 3:**
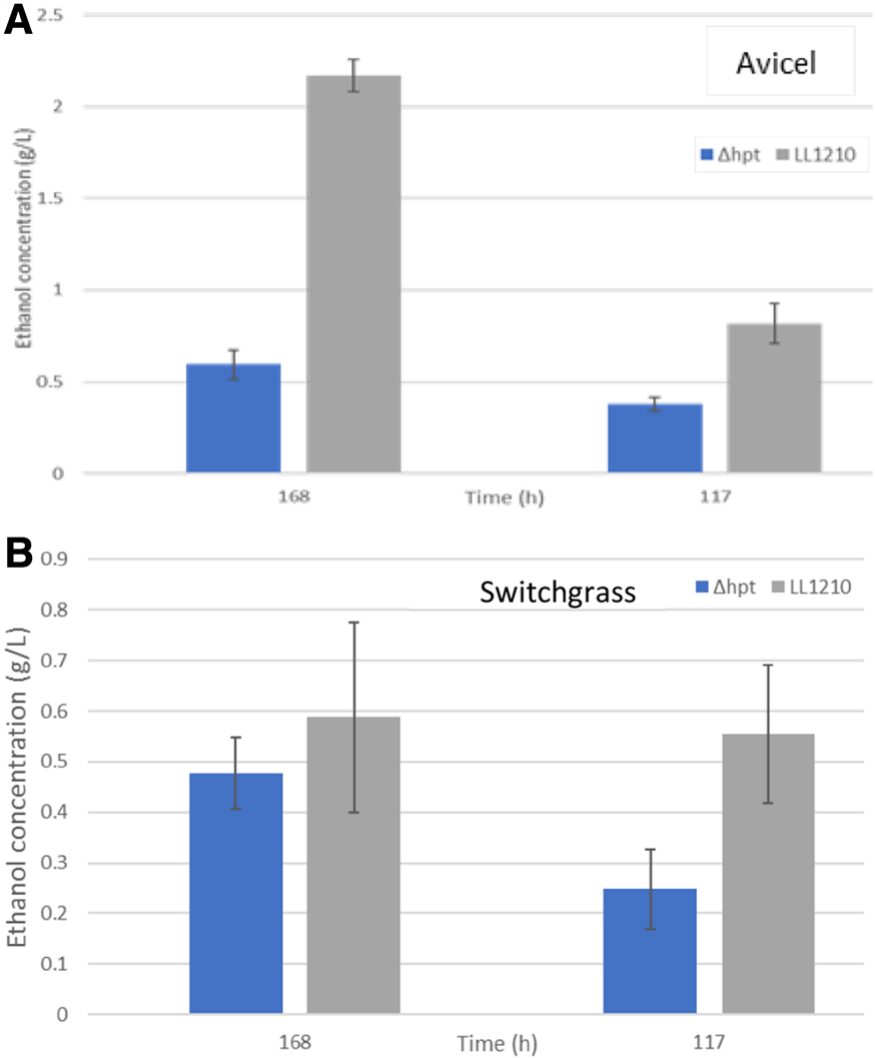
Ethanol production by *C. thermocellum* strain Δ*hpt* and strain LL1210 with **a** Avicel or **b** switchgrass as the substrate

Strain LL1210, with deletions in key metabolic genes that enhance ethanol titers and its ability to utilize cellulose as a carbon and energy source, has been used for pH controlled bioreactor studies over a 140 h incubation (Tian et al. 2016). The production of ethanol using either Avicel or switchgrass as substrates (Fig. 3a, b respectively) for strains Δ*hpt* and LL1210 are shown for two CBP plate incubation time points. Strain LL1210 produced higher ethanol titers compared to strain Δ*hpt*, consistent with expectations and earlier reports. Plate titers for ethanol are lower than in pH-controlled bioreactors and lower ethanol concentrations were achieved for the model substrate. While this should be acknowledged, the primary purpose of these experimental HTP screenings is to resolve differences in feedstock or microorganism properties, such that a HTP plate model may replace the more expensive bioreactor models. Therefore, more interesting biomass and microbial variants will require further testing and validation in larger scale fermentations.

Further studies are needed to assess the plate assay in combination with other analytical methods, such as quantitative saccharification analysis of residual biomass, and to what extent pooling of wells is needed to determine the extent of hydrolysis and fermentation. The effect of biomass on bioethanol can be further investigated by varying the amount of biomass in each well. In this study, controls were included to ensure that measured metabolite products were a product of fermentation of biomass, and not compromised by background interference from either the biomass or inoculum. The effect of evaporative loss from the plate, as pointed out by Elliston et al. (2015), also needs to be investigated. Though this study used switchgrass as lignocellulosic biomass for production of bioethanol, additional information on CBP assays could be obtained by using additional LCBs such as poplar. Greater diversity of LCB will allow for better characterization and diversity of CBP for bioethanol production. Use of multiple microbial strains in this screening platform provides a more comparative picture of microbial specificity. The rapid high throughput assay offers technical advantages over the flask-based procedures normally used to assay bioethanol production from lignocellulosic biomass. The methodology is highly applicable to current, large-scale screening efforts designed to improve bioethanol production. Similarly, this methodology will greatly facilitate parallel efforts to select appropriate lignocellulosic feedstocks for biomass conversion and to resolve the techno-economic constraints currently associated with LCB pretreatment processes. This methodology could be used to replace large shake flask reactions with comparatively fast 96-well plate CBP assays allowing for HTP experimentation. Furthermore, this research has practical uses in the bio refining of biomass substrates for second generation biofuels and novel bio-based chemicals by allowing HTP CBP screening, which should allow selected samples to be scaled up or studied in more detail.

## Conclusions

Proof of concept that a 96 well microplate can be used to assay lignocellulosic biomass for bioethanol production in a high-throughput Consolidated Bioprocessing configuration has been demonstrated.
